# Study Protocol for the COVID-19 Pandemic Adjustment Survey (CPAS): A Longitudinal Study of Australian Parents of a Child 0–18 Years

**DOI:** 10.3389/fpsyt.2020.555750

**Published:** 2020-08-31

**Authors:** Elizabeth M. Westrupp, Gery Karantzas, Jacqui A. Macdonald, Lisa Olive, George Youssef, Christopher J. Greenwood, Emma Sciberras, Matthew Fuller-Tyszkiewicz, Subhadra Evans, Antonina Mikocka-Walus, Mathew Ling, Robert Cummins, Delyse Hutchinson, Glenn Melvin, Julian W. Fernando, Samantha Teague, Amanda G. Wood, John W. Toumbourou, Tomer Berkowitz, Jake Linardon, Peter G. Enticott, Mark A. Stokes, Jane McGillivray, Craig A. Olsson

**Affiliations:** ^1^Deakin University, Centre for Social and Early Emotional Development, School of Psychology, Geelong, VIC, Australia; ^2^Population Health, Murdoch Children’s Research Institute, Melbourne, VIC, Australia; ^3^Department of Paediatrics, University of Melbourne, VIC, Australia; ^4^The National Drug and Alcohol Research Centre, The University of Sydney, Sydney, NSW, Australia; ^5^Centre for Educational Development, Appraisal and Research, University of Warwick, Coventry, United Kingdom; ^6^Aston Neuroscience Institute and School of Life and Health Sciences, Aston University, Birmingham, United Kingdom

**Keywords:** COVID-19 pandemic, mental health, parenting, mother, father, child mental health, couple conflict, family functioning

## Abstract

**Background:**

The COVID-19 pandemic presents significant risks to the mental health and wellbeing of Australian families. Employment and economic uncertainty, chronic stress, anxiety, and social isolation are likely to have negative impacts on parent mental health, couple and family relationships, as well as child health and development.

**Objective:**

This study aims to: (1) provide timely information on the mental health impacts of the emerging COVID-19 crisis in a close to representative sample of Australian parents and children (0–18 years), (2) identify adults and families most at risk of poor mental health outcomes, and (3) identify factors to target through clinical and public health intervention to reduce risk. Specifically, this study will investigate the extent to which the COVID-19 pandemic is associated with increased risk for parents’ mental health, lower well-being, loneliness, and alcohol use; parent-parent and parent-child relationships (both verbal and physical); and child and adolescent mental health problems.

**Methods:**

The study aims to recruit a close to representative sample of at least 2,000 adults aged 18 years and over living in Australia who are parents of a child 0–4 years (early childhood, N = 400), 5–12 years (primary school N = 800), and 13–18 years (secondary school, N = 800). The design will be a longitudinal cohort study using an online recruitment methodology. Participants will be invited to complete an online baseline self-report survey (20 min) followed by a series of shorter online surveys (10 min) scheduled every 2 weeks for the duration of the COVID-19 pandemic (i.e., estimated to be 14 surveys over 6 months).

**Results:**

The study will employ post stratification weights to address differences between the final sample and the national population in geographic communities across Australia. Associations will be analyzed using multilevel modeling with time-variant and time-invariant predictors of change in trajectory over the testing period.

**Conclusions:**

This study will provide timely information on the mental health impacts of the COVID-19 crisis on parents and children in Australia; identify communities, parents, families, and children most at risk of poor outcomes; and identify potential factors to address in clinical and public health interventions to reduce risk.

## Introduction

The World Health Organization declared COVID-19 a pandemic on the 11^th^ of March 2020. Consistent with government responses around the world, the Australian federal and state governments introduced an increasingly strict regime of social distancing/isolation measures to slow the rate of infection (1). These measures may present significant risks to the population, over and above the health threat associated with COVID-19 (2, 3). Findings from a cross-sectional study of 2,077 participants recruited in 22 countries in late March and early April 2020 indicated that adult mental health symptoms at that time were elevated compared to historical norms, with participants’ concern about the COVID-19 pandemic and loss of employment associated with higher levels of mental health problems (4). It is as yet unknown what the full impact of the pandemic will be on Australian families.

The COVID-19 pandemic represents an unprecedented confluence of risk in Australia and globally in this century, including: (1) a high level of uncertainty in regard to the parameters, time frames, and outcomes of the pandemic; (2) high rates of unemployment or underemployment, and housing and economic uncertainty; (3) threat to, or reduction of protective factors, such as social and community connection, physical activity, access to greenspace, and other co-curricular activities; and restricted access to clinical, community, family, and other supports and services; (4) increased pressure on parents to supervise and/or home-school children while juggling working from home; and (5) risk associated with being ‘locked in’ with family members in close quarters. It is unknown what effect the combination of these risks may have on the population of parents. However, each of these factors have an evidence-base demonstrating potential risks to adult and child mental health and wellbeing (5–16). There is evidence showing increased risks of mental health problems, drug and alcohol use, and family violence during and after crisis events and disasters (5–7, 17). Job loss, employment uncertainty, and difficulties in juggling work and family roles are associated with increases in parent mental health problems, couple conflict, and child mental health problems (8–16). Finally, there is evidence that quarantine is associated with a range of negative psychological outcomes including post-traumatic stress symptoms, confusion, and anger (3).

It is important to understand the experiences and consequences of the COVID-19 pandemic for all Australian families in order to plan for appropriate intervention and support, both during and after the pandemic period. However, the pandemic is likely to have a disproportionate effect on vulnerable parents and families. There is an urgent need to understand the impact for families with pre-existing risk factors to ensure that any public health interventions are appropriately tailored to these subgroups (2). Mental health problems are highly prevalent, affecting approximately one in five adults in Australia (18). It will be important to understand how adults with a pre-existing mental health problem or other personal vulnerabilities, such as difficulties in managing relationships and emotions (i.e., attachment insecurity and difficulties regulating emotions), respond to the COVID-19 pandemic. In addition, approximately one in seven children and adolescents experience a mental health or neurodevelopmental disorder, such as attention-deficit/hyperactivity disorder or autism spectrum disorder, equating to about 560,000 young people in Australia (19). In Australia and other nations, child mental health problems are clustered in places of disadvantage (20). To88 date, there is limited evidence as to how place‐based epidemic management affects disadvantaged communities. This study represents an important opportunity to understand how Australian communities and families affected by such conditions adjust to a global pandemic. Further, adults with chronic physical health conditions (such as diabetes, cardiovascular disease, and autoimmune conditions) are also at increased risk of negative outcomes *via* the potential for (1) more serious illness outcomes (21), (2) exacerbation of their health condition(s) caused by psychosocial stress and depression (22–24); and increased risk of infection in context of immune system impairment (25) or immunosuppressive treatments (26).

This study will investigate the impact of COVID-19 on the health and wellbeing of parents, children, and families. Specifically, the study will examine:

1. The extent to which the developing COVID-19 pandemic over time is increasing risk for:
  - Parent mental health problems, poor wellbeing, loneliness, and alcohol use;
  - Parent-parent (verbal and physical conflict) and parent-child relationship problems;
  - Child and adolescent mental health problems.
2. Whether some families and communities have a higher risk of experiencing these problems over time compared to other families, including:
  - Families with a member with a pre-existing mental health problems, attachment insecurity, and/or recent stressful life events;
  - Families living with or supporting those with a physical health condition or disability;
  - Families experiencing financial strain, crisis-associated job loss, and/or on low incomes or government benefits.
3. Whether there are modifiable factors that moderate families’ experience of risk over time, that could be targeted to strengthen families during and after the crisis, including:
  - Individual: promoting emotion-regulation, sleep quality, physical activity, and healthy screen-use;
  - Couple: promoting supportive relationships and constructive management of conflict; Familial: promoting nurturant parenting and positive familial communication.

## Methods

### Design

This is a longitudinal cohort study of Australian parents of a child aged 0–18 years. The study comprises two sets of online surveys scheduled on a regular basis for the duration of the COVID-19 pandemic. The surveys include:

1. A repeated baseline survey (20 min) scheduled at baseline and at 3-month intervals and
2. A brief longitudinal survey (10 min) scheduled every 2 weeks.

The time frame of the study will extend across the duration of the social distancing measures implemented by the Australian federal and state governments to manage the COVID-19 pandemic in Australia. The federal government released a statement estimating that the likely time frame will be a period of six months from March 2020 to September 2020 (27). The regularity and time-frame of the longitudinal surveys will be reviewed every 2–3 months to ensure that benefits of regular follow-up are weighed against potential for participant burden and fatigue.

### Eligibility

Participants will be eligible to participate if they are an Australian resident, 18 years or over, and are a parent of a child aged 0–18 years. Survey information and advertisements will be written in English, so it is expected that people with adequate English fluency will complete the survey.

### Recruitment

Parents will be recruited *via* paid and unpaid social media advertisements. A range of methods will be used to target specific groups to increase the representativeness of the sample (e.g., targeting *via* postcodes and demographic factors). The style and wording of advertisements is important in determining recruitment success. Consistent with prior research, this study will employ advertisements that: (1) refer to research; include the Deakin University affiliation, refer to the incentive (as detailed below), and are written in engaging yet plain language (28).

Participants will primarily be recruited *via* the social media platform, Facebook, given demonstrated success in recruiting hard-to-reach populations on this platform (29, 30). A project ‘business’ Facebook page will be established to maintain contact with participants, affiliate organizations, and the wider public. The page will be monitored regularly by project staff and any content/comments deemed inappropriate or offensive will be promptly removed. Both paid and unpaid recruitment strategies on Facebook will be used in the current study. Unpaid strategies will include making contact with established interest groups, parenting groups, and organizations on Facebook *via* the project Facebook page and/or Deakin University email (i.e., where email addresses are provided), and requesting that these sites endorse our project by posting the project advertisement so that it is visible to their group members. Paid strategies will involve using Facebook’s systems to target recruitment to specific sub-populations *via* demographic variables (e.g., parents of children 0–18 years; fathers, remote/regional postcodes, and parents speaking a language other than English), posting paid advertisements on all available platforms, including Facebook and Instagram. We will also use other social media platforms (e.g., Reddit, Twitter, Instagram, and WhatsApp) following the same protocols to post both paid and unpaid advertisements as per our current use of Facebook.

### Expected Sample Size

The study aims to recruit a minimum of 2,000 parents of a child 0–4 years (early childhood, N = 400), 5–12 years (primary, N = 800), or 13–18 years (secondary, N = 800).

### Procedures

#### Baseline Survey

The advertisements used for recruitment will contain a web hyperlink which will direct participants to an initial Qualtrics survey website. The landing page for the survey will contain a brief description of the purpose of the research. On the next page, participants will be asked two eligibility questions, checking that they are a parent of a child 0–18 years and that they currently live in Australia. If participants are not eligible to participate, they will be directed out of the survey with an explanation of the eligibility criteria. Eligible participants will then be presented with a Plain Language Statement and Online Consent form available for download as a PDF document. On this page, participants will be asked to check a box that confirms that they have read the Plain Language Statement, which they understand its contents, and consent to participate in the study. Participants will then be asked to provide contact information with details of their first name, phone number, and email address. A brief explanation will be provided that this information will assist the research team in contacting the participants for the follow-up survey, sending reminders, and contacting winners of the monthly prize draw. On completion of the baseline survey, participants will be automatically allocated a unique ID number, which will be embedded in their subsequent surveys to identify them and link their data.

An invitation (and web link) will be included at the end of the Qualtrics baseline survey inviting the potential participant to ‘friend’ the CPAS page on Facebook. This is intended as a strategy to maximize participant retention rates and promote participant connectedness to the study. Facebook allows a stable means of communication where participants can be contacted for future time points of the study regardless of changes in contact details. This request would be a means of keeping the study in the minds of participants as study updates and news would appear on the participant’s own Facebook ‘News Feed.’ Only one email request would be sent with no follow-ups, even if the request is declined or ignored. No changes would be made to the previously approved Facebook privacy settings.

#### Fortnightly Longitudinal Survey

Participants will be re-contacted every 2 weeks after completion of the baseline survey *via* an automated email invitation. Participants will be recruited on a rolling basis to maximize reach and sample size. Regardless of whether a participant responds in a given week, participants will remain on the active list and will continue to receive survey invites and reminders. All emails to participants will contain an opt-out link with two options: to opt-out from the survey or to opt-out of the study entirely.

#### Participant Reminders

Participants who open the baseline survey, consent to participate, and who have provided their contact details but did not complete the full version of the online baseline survey will be sent an email reminder about completing the survey 24 hours later. Participants will be sent an email reminder 24 hours after each fortnightly longitudinal survey is sent. If participants have not completed a survey or made contact with the study team over a period of three consecutive surveys, the team will use a range of methods to attempt to re-engage participants in the study. This may include sending an additional follow-up email, sending an SMS reminder and/or calling the participant on their mobile phone number, or contacting the participant *via* Facebook (refer to section *Facebook Tracing*, below). We will limit all contacts to a maximum of 1 direct contact (i.e., involving communication from the participant) within a week, *via* email, SMS, or voicemail message. In order to understand reasons for participant drop out, we will ask participants two brief questions when making contact *via* phone, a question asking about the participants’ reasons for not completing follow-up surveys (“Day to day life is very busy”; “Want to complete but forget or never get around to it”; “Change in your circumstances—decrease in job hours/loss job; increase in job hours, gained employment, started studying, stopped studying, change in caregiving responsibilities”; “Lost interest in the survey”; “Other”) and a question assessing participants’ level of functioning (“Compared to when you first completed the survey in April – this was around the beginning to middle of the most restrictive period in Australia – would you say you are going about the same, better, or worse right now? “).

#### Facebook Tracing

For participants whom we are not able to contact (no email response or a return to sender email; and no evidence that we reached the correct participant’s phone—i.e., no identifying voicemail message or the number was disconnected), we propose attempting contact *via* Facebook. Facebook searches will be conducted to generate evidence from which to identify participants. Only publicly available information will be viewed based on information publicly visible on users’ profiles, “Liked Pages,” “Groups,” or “Check-ins” to verify the location of the participant, compared to their last known residential address. If the study team has strong evidence to link a Facebook user with the identity of a previous participant, participants will be contacted through Facebook Messenger.

#### Remuneration for Participation

Research has shown benefits associated with the use of incentives in social media recruitment *via* Facebook (28, 31). Participants will be entered into a prize draw for 1 of 10 AU $50 online gift vouchers if they have completed at least one survey for every month of the survey. We have estimated vouchers based on a study of six months’ duration (6 prize draws, 10 vouchers offered at each draw, a total of 60 vouchers).

#### Consent

Consent will be obtained at baseline. Participants will also complete separate (optional) consent to be contacted for future research participation. Participants will be informed that they are under no obligation to participate and advised that they are free to withdraw at any time without consequences.

#### Data Management

Study data will be managed using Qualtrics, hosted at Deakin University (32). Data will be downloaded from the Qualtrics server on a weekly basis and stored on servers maintained by Deakin University.

### Measures

**Table 1** provides an outline of study measures. Where possible, measures will be harmonized with the Longitudinal Study of Australian Children (LSAC), a population representative sample of Australian families. LSAC includes two cohorts of children and families recruited in 2005 and followed biennially on an ongoing basis (altogether, N = 10,000 at baseline) (52).

**Table 1:** Overview of measures included in the COVID-19 Pandemic Adjustment Survey (CPAS).

**Construct**	**Measure (items)**	**Baseline/fortnightly**
Demographics	Family demographics and socio-economic questions	Baseline
COVID-19	COVID-19 factors (adapted from the CoRonavIruS Health Impact Survey (CRISIS) V0.1. (33)	Baseline-fortnightly
**Parent factors**		
** **Well-being	Personal well-being index adult (34) (seven items)	Baseline-fortnightly
** **Personality	Introvert/extrovert	Baseline
** **Mental health	Depression and anxiety scale (DASS) 21-item version (35)	Baseline-fortnightly
** **	Mental or physical health diagnosis	Baseline
** **Emotion regulation	Difficulties in emotion regulation scale-16 item version (36)	Baseline
** **Positive affect	Positive and negative affect schedule short form (37) (five items)	Baseline
** **Physical health	Physical activity (1 item) from the Longitudinal Study of Australian Children (LSAC)^a^; sleep (one item) from LSAC	Baseline-fortnightly
** **Substance use	Alcohol consumption (1 item) from LSAC; cigarette smoking (one item) from LSAC	Baseline-fortnightly
** **Adult attachment	Experiences in close relationships scale–relationship structures (ECR-RS) (38) (nine items)	Baseline
** **Resilience	Brief resilience scale (BRS) (39) (six items)	Baseline
** **Loneliness	UCLA loneliness scale (40) (six items)	Baseline-fortnightly
** **Utopian thinking	Utopian thinking (one item)	Baseline
**Family functioning**		
** **Family expressiveness	Adapted short-form of the self-expressiveness in the family questionnaire (41) (11 items)	Baseline-fortnightly
** **Stressful life events	Stressful life events over the past 12 months (42) (nine items)	Baseline
** **Couple conflict	Argumentative relationship scale used in LSAC (43) (five items)	Fortnightly
** **		
** **Relationship quality	Perceived relationships quality component (PRQC) questionnaire (44) (baseline survey, six items; fortnightly survey, one item only).	Baseline
** **Social support	Social support (1 item) from LSAC; social provisions scale (one item) (45); secure base characteristics scale (one item) (46)	Baseline-fortnightly
** **Neighbourhood disadvantage	Postcodes used to derive the Socio-Economic Indexes for Areas (SEIFA) advantage and disadvantage (47)	Baseline
** **Parenting	Interpersonal mindfulness in parenting (IEM-P) (48) (three items); Emotion-focussed parenting (three items); parenting irritability from LSAC (49) (five items)	Baseline-fortnightly
**Child outcomes**		
** **Physical health	Global child health	Baseline-fortnightly
** **Child diagnosis	Professional diagnosis or treatment	Baseline
** **Mental health	The short mood and feelings questionnaire (SMFQ) (50) (13 items); modified brief spence children’s anxiety scale (51) (four selected items); SNAP-IV 26-item parent rating scale, opposition/defiance (four selected items). Irritability (one item) and loneliness (one item) adapted from the CoRonavIruS Health Impact Survey (CRISIS) [28]	Baseline-fortnightly
** **Mood	Child mood (eight items) (fortnightly survey only)	
** **Physical health	Physical activity (one item) adapted from LSAC; sleep pattern and regularity (two item) from LSAC	Baseline-fortnightly
** **Screen-time	Screen time (two items) from LSAC	Baseline-fortnightly
**Intervention**		
** **Interest in online interventions	Likelihood of using an online intervention (one item)	Baseline-fortnightly
** **Type online intervention	Likelihood of using self-guided or therapist assisted online mental health intervention (two items)	Baseline-fortnightly

The Longitudinal Study of Australian Children (LSAC) is a population-representative government-funded study comprising of two cohorts of children and their families recruited in 2005 and followed biennially (together, N = 10,000).

#### Demographic and COVID-19 Variables

##### Identifiable Information (First Baseline Survey Only)

First name, email address, mobile number, and postcode.

##### Demographics (Baseline Survey Only)

*About adult:* Age, gender, country of birth, Aboriginal and Torres Strait Islander status, language other than English spoken at home, education, relationship status, whether living with partner, and number of children in the household. Demographics prior to COVID-19: employment, study, household income, source of income, and shortage of money. Items about housing: type of dwelling, owned or rented, number of bedroom, number of people living in house, satisfaction with quality of housing, and access to private outdoor space at current home.

*About partner:* Gender; partner’s relationship to child, employment, and education.

*About child:* Age, gender, and education setting.

#### COVID-19 Factors (Baseline and Fortnightly Survey)

Items adapted from the CoRonavIruS Health Impact Survey (CRISIS) V0.1 (33).

*Household:* COVID-19 diagnosis, test result, or symptoms.

*About adult:* Participant or family members affected by COVID-19 (fallen ill, hospitalized, self-quarantine, and passed away), financial problems or housing and food insecurity related to COVID-19, working from home, frequency and type of contact with work colleagues, impact on family life, food/medical shortages, use of media, feelings and attitudes about COVID-19, impact of COVID-19 on family life (short-answer question, “How has COVID-19 affected your family life?”), coping strategies (short-answer question, “What strategies are helping you to stay calm in the current situation?”), frequency of use of news sources (newspapers, television, social media, radio, rated on 6-point scale from ‘not at all’ to ‘multiple times per day’), appraisals of COVID-19 as a serious health risk, and whether likely to catch COVID-19 (both rated on a 7-point scale from ‘strongly disagree’ to ‘strongly agree’).

*About child:* Presence of a daily routine at home, time outside home (going to stores, parks, etc.), child’s relationship quality with their friends (rated on 5-point scale from ‘a lot worse’ to ‘a lot better’). Whether school classes are running on campus, school attendance on campus or online. For children home-schooling: whether child home with parent while they work, child’s internet/computer access at home, whether they have assignments to complete from home, amount of school work completed each day, and parents rating of how well they are managing child’s home learning (4-point scale from ‘very poorly’ to ‘very well’).

#### Adult Outcomes

##### Wellbeing (Baseline and Fortnightly Survey)

*Personal Wellbeing Index* (34) (seven items). seven domains: standard of living, personal health, achieving in life, personal relationships, personal safety, community-connectedness, and future security. Example item: “How satisfied are you with … your standard of living?” Rated on a 11-point scale from ‘no satisfaction at all’ to ‘completely satisfied’.

##### Personality (Baseline Survey Only)

*Introvert/extrovert* (one item, designed for the current study) “Do you consider yourself an introvert?” rated on a 7-point scale from ‘introvert’ to ‘extrovert.’

##### Mental Health (Baseline and Fortnightly Survey)

*Depression and Anxiety Scale (DASS)* 21-item version (35). Three subscales: depression, stress, and anxiety (seven items each). Example item: “I found it hard to wind down.” Rated on a 4-point scale from ‘did not apply to me at all’ to ‘applied to me very much, or most of the time.’

##### Mental or Physical Health Diagnosis (Baseline and One Fortnightly Survey Only)

One item (baseline): “Have you ever had a professional diagnose or treat you for a mental or physical health condition? What was the condition?” One item (presented at one fortnightly survey): Have you ever been treated or diagnosed for any of the following chronic physical conditions by a health professional? Ulcerative Colitis, Crohn’s disease, endometriosis, cardiovascular disease (e.g., coronary heart disease, stroke, and heart failure); hypertension (clinically high blood pressure), type 1 diabetes, type 2 diabetes, and other.

##### Emotion Regulation (Baseline Survey Only)

*Difficulties in Emotion Regulation Scale-16 Item Version* (36) (16 items). Five subscales: strategies, non-acceptance, impulse control, goals, and clarity. Example item: “I have difficulty making sense out of my feelings.” Rated on a 5-point scale from ‘almost never’ to ‘almost always.’

##### Positive Effect (Baseline Survey Only)

*Positive Affect Subscale* from the positive and negative effect schedule short form (37) (five items). Example item: “Thinking about yourself in the past 4 weeks, about how often did you feel … alert?” Rated on a 5-point scale from ‘very slightly or not at all’ to ‘extremely.’

##### Physical Health (Baseline and Fortnightly Survey)

*Physical activity* (one item) from the Longitudinal Study of Australian Children (LSAC). Item: “About how many days each week do you do at least 30 min of moderate or vigorous physical activity (like walking briskly, riding a bike, gardening, tennis, swimming, running, etc)?” Rated from 1 to 7 days.

*Sleep* (one item) from LSAC. Item: “During the past month, how would you rate your sleep quality overall?” Rated on a 4-point scale from ‘very good’ to ‘very bad.’

##### Substance Use (Baseline and Fortnightly Survey)

*Alcohol consumption* (one item) from LSAC. Item: “How often do you have a drink containing alcohol?” Rated on a 7-point scale from ‘never’ to ‘every day.’

*Cigarette smoking* (one item) from LSAC. Item: “How often do you smoke cigarettes?” Rated on a 3-point scale from ‘do not smoke at all’ to ‘at least once a day.’

##### Adult Attachment (Baseline Survey Only)

*Experiences in Close Relationships Scale*–*Relationship Structures* (ECR-RS) (38) (nine items). Two subscales: attachment anxiety and attachment avoidance. Example item: “It helps to turn to people in times of need.” Rated on a 7-point scale from ‘strongly disagree’ to ‘strongly agree.’

##### Resilience (Baseline Survey Only)

Brief resilience scale (BRS) (39) (six items). Example item: “I tend to bounce back quickly after hard times.” Rated on a 5-point scale from ‘strongly disagree’ to ‘strongly agree.’

##### Loneliness (Baseline and Fortnightly Survey)

UCLA loneliness scale (40) (six items). Example item: “I lack companionship.” Rated on a 4-point scale from ‘never’ to ‘always.’

##### Utopian Thinking (Baseline and Fortnightly Survey)

Utopian thinking (one item). Item: “I often think about what an ideal society might look like.” Rated on a 7-point scale from ‘strongly disagree’ to ‘strongly agree.’

#### Family, Couple, and Parenting Outcomes

##### Family Expressiveness (baseline and fortnightly survey)

*Adapted short-form of the Self-Expressiveness in the Family Questionnaire* (41) (11 items were selected according to a consensus of three independent expert ratings evaluating item relevance in relation to the COVID-19 pandemic). Two subscales: positive and negative expressiveness. Example item: “Showing contempt for another’s actions.” Rated on a 9-point scale from ‘not at all frequently in my family’ to ‘very frequently in my family.’

##### Stressful Life Events (Baseline Survey Only)

*Stressful life events over the past 12 months* (42) (eight items). Example items: “In the last year, have any of the following happened to you (or your partner)? You became pregnant or had a baby; You moved house.” Items rated Yes/No.

##### Couple Conflict (Baseline and Fortnightly Survey)

*Argumentative Relationship Scale used in LSAC* (43) (five items). Example item: “How often do you and your partner disagree about basic household issues?” Rated on a 5-point scale from ‘never’ to ‘always.’

##### Relationship Quality (Baseline and Fortnightly Survey)

*Perceived Relationships Quality Component* (PRQC) *Questionnaire* (44) (six items measured in baseline survey and one item in fortnightly survey). Example item (and item in fortnightly survey): “How satisfied are you with your relationship?” Rated on a 7-point scale from ‘not at all’ to ‘extremely.’.

##### Social Support (Baseline and Fortnightly Survey)

Social support (one item) from LSAC. Item: “Overall how do you feel about the amount of support or help you get from family or friends living elsewhere?” Rated on a 4-point scale from ‘I get enough help’ to ‘I don’t get any help at all’ and ‘I don’t need any help.’

*Social Provisions Scale* (45) (one item selected). Item: “When I am feeling stressed about a new or unknown situation, I can rely on my partner to comfort me.” Rated on a 7-point scale from ‘strongly disagree’ to ‘strongly agree.’

*Secure Base Characteristics Scale* (46) (one item selected). Item: “My partner encourages me to draw on my skills and abilities to deal with challenges”. Rated on a 7-point scale from ‘strongly disagree’ to ‘strongly agree.’

##### Neighborhood Disadvantage (Baseline Survey Only)

Postcodes used to derive neighborhood disadvantage according to the Socio-Economic Indexes for Areas (SEIFA) advantage and disadvantage (47).

##### Parenting (Baseline and Fortnightly Survey)

*Interpersonal Mindfulness in Parenting* (IEM-P) (48) (three items). Example item: “When I’m upset with my child, I notice how I am feeling before I take action.” Rated on a 5-point scale from ‘almost never’ to ‘almost always.’

*Emotion-Focused Parenting* (three items). Example item: “When my child experiences strong emotions (sad, angry, scared), I connect with them and provide comfort and support.” Rated on a 5-point scale from ‘almost never’ to ‘almost always.’

*Parenting Irritability* (five items) from LSAC. Example item: “In the past 6 months, how often would you say … I have raised my voice with or shouted at this child.” Rated on a 10-point scale from ‘not at all’ to ‘all the time.’

#### Child Outcomes

##### Physical Health (Baseline and Fortnightly Survey)

*Global child health from LSAC*. Item: “In general, is your child’s current health…” Rated on a 5-point scale from ‘excellent’ to ‘poor.’

##### Child Diagnosis (Baseline Survey Only)

*Professional diagnosis or treatment* (one item). Item: “Has your child ever been diagnosed or treated for any of the following by a health professional?” Response options (rated Yes/No): ADHD; autism, Asperger’s, other autism spectrum; oppositional defiant or conduct disorder; speech or language disorder; head injury, epilepsy, seizure(s), febrile convulsions; disability; and other (free text).

##### Mental Health (Baseline and Fortnightly Survey)

*The Short Mood and Feelings Questionnaire* (SMFQ) (50) (13 items). One scale: Depression. Example item: “Your child felt miserable or unhappy.” Rated on a 3-point scale from ‘not true’ to ‘true.’

*Modified Brief Spence Children’s Anxiety Scale* (51) (four selected items). One scale: Anxiety. Example item: “My child worries about things.” Rated on a 4-point scale from ‘never’ to ‘always’.

*Swanson, Nolan, and Pelham –IV Questionnaire (SNAP-IV)* (53) *Parent Rating Scale*, *Opposition/Defiance Subscale* (four selected items). Example item: “Often actively defies or refuses adult requests or rules” Rated on a 4-point scale from ‘not at all’ to ‘very much.’

*Loneliness* (one item) adapted from the CoRonavIruS Health Impact Survey (CRISIS) (33). Item: “During the past 2 weeks, how lonely has your child been?”

*Irritability* (one item) adapted from the CoRonavIruS Health Impact Survey (CRISIS) (33) Item: “During the past 2 weeks, how irritable or easily angered has your child been?”

*Child mood* (eight items) (fortnightly survey only). Item: “Please indicate below how your child is feeling: happy, sad, content, bored, excited, anxious, alert, tired.” Rated on a 11-point scale from ‘not at all’ to ‘very much.’

##### Physical Health (Baseline and Fortnightly Survey)

*Physical activity* (one item) adapted from LSAC. Item: “About how many days each week does your child do at least 30 min of moderate or vigorous physical activity (like walking briskly, riding a bike, swimming, running, etc)?” Rated from 1 to 7 days.

*Sleep pattern* (one item) from LSAC. Item: “How much is your child’s sleeping pattern or habits a problem for you?” Rated on a 4-point scale from ‘not a problem at all’ to ‘a large problem.’

*Sleep regularity* (one item) from LSAC. Item: “Does the study child go to bed at regular times?” Rated on a 5-point scale from ‘never’ to ‘always.’

##### Screen-Time (Baseline and Fortnightly Survey)

*Screen time* (two items) adapted from LSAC. “About how many hours on a typical weekday does your child watch TV or videos at home not for educational purposes? (e.g., YouTube, Instagram, TikTok, streaming services such as Netflix).” Rated on a sliding scale from 1 to 24 hours.

#### Intervention Willingness (Baseline and Fortnightly Survey)

*Online intervention* (three items). Items: “The COVID-19 pandemic and the associated measures to increase social distancing have caused many people to feel stressed and worried. How likely would you be to use an online or smartphone intervention for the following reasons: Mental health support for yourself, mental health support for your child, and parenting support.” Rated on a 5-point scale from ‘not at all’ to ‘extremely likely.’

*Mental health intervention* (two items). Items: “Should you experience a mental health difficulty in the future, how likely are you to use a … Self-guided internet- or smartphone-app based treatment program? Therapist-assisted internet- or smartphone-app based treatment program?” Rated on a 5-point scale from ‘extremely likely’ to ‘extremely unlikely.’

### Analysis Approach

#### Quantitative Data

##### Data Preparation

Data will be prepared in Stata version 16 (54). Missing data will be addressed using either full information maximum likelihood estimation or multiple imputation by chained equations, depending on the analysis. Both methods rely on the untestable assumption that missingness is ignorable. Sensitivity analyses (e.g., in the form of selection models or pattern mixture models) will be conducted to evaluate impact of violation of this assumption on modeled results (55).

#### Data Analysis

Analyses will be conducted in Stata version 16, or where relevant, in Mplus version 8 (56). The planned approach for testing Aims 1–3 is outlined below. Where relevant, all associations will be investigated in unadjusted analyses, and then in adjusted analyses, the latter controlling for the baseline effects of factors known to be associated with adult socio-emotional adjustment (gender, age, health, family demographic factors). Decisions about the inclusion of specific covariates in each model will be made using directed acyclic graphs (DAGs) (57). Associations will be analyzed using multilevel modeling in either a latent variable or mixed effects framework to: (i) account for the clustered nature of time points within individuals while (ii) also modeling between-individual differences in rate of change over time. In these models, we will regress an outcome (e.g., mental health) on to ‘time,’ any moderator variables of interest, and background covariates. We anticipate ‘time’ being treated as a continuous predictor in all models (with the baseline time-point coded as 0 and then numbered consecutively), but we will also consider treating ‘time’ as a categorical variable with discrete categories of time demarcated by important events that may occur during the pandemic window. The influence of potential moderators on the relationship between these associations will be investigated by including interaction terms (e.g., moderator x time).

#### Population Weighting

We will use post-stratification weights, generated through a raking approach (58) to compensate for differences between the final sample and the national population across geographic community clusters, parent age, gender, educational attainment, and country of birth (Australia/New Zealand versus other). We will ensure that strata sample sizes are large enough to not unduly influence the overall results.

#### Power Calculation

Power is demonstrated for our key analyses involving within person relationships during the longitudinal study. Given the clustered nature of the study of time points nested within participants, the Effective Sample Size (ESS) for the study is given by ESS = nm/(1+(m-1)ρ) (59), where n = number of participants aiming to be recruited, m = number of data points per cluster, and ρ = the within cluster correlation. Based on a 6-month window of data collection and fortnightly assessments (estimated 14 assessments), the smallest sample of 400 participants (parents of a child 0–4 years) has an ESS = 746 assuming a conservative within cluster correlation of ρ = .5. Using Monte Carlo simulation (10,000 draws) in Mplus 8, an ESS = 746 would provide 98.2% power to detect a true effect of interest (e.g., time related change in parent mental health problems) of even small magnitude (β = .14, representing just ~2% extra variance accounted for in the outcome above a base level of ~10% by other variables in the model; at α = .05, two-tailed). Thus, the study is well powered for even small true effects of interest. Note that even if participants only complete two of the assessments (ESS = 533), this would still provide 92.7% power to detect the above-mentioned effect for our smallest age stratified group (parents of a child 0–4 years). Additionally, for any between person relationships (e.g., differences between families), even the minimum sample size of 400 would provide 84.8% power to detect effects of the above-mentioned size. Thus, the study is well powered.

### Qualitative Analysis

Qualitative data will be analyzed using thematic analyses to determine the common themes that arise from the participant answers to the two short-answer questions posed regarding parent’s coping strategies and impact of COVID-19 on family life (60), Thematic analysis is a method of analyzing qualitative data that is focused on identifying, examining, and recording major patterns or themes in the data.

### Research Study Administration

#### Ethics Statement

The current study has been approved by the Deakin University Human Ethics Advisory Group (Project number: HEAG-H 52_2020).

#### Ethical Issues

We use brief screening measures to assess adult and child functioning. These measures are routinely used in population-level, large scale, longitudinal surveys, but are not designed to collect clinical information, thus the scales cannot be used to diagnose physical or mental health conditions. Participants will be provided with a Plain Language Statement that outlines the key constructs assessed in the study, reminds participants they can withdraw at any time, and provides information on where participants can seek help if any of the questions do cause them discomfort or distress. It will be possible for participants to skip any of the questions/items in the survey, and to facilitate this, none of the special case assessment items on the online survey form will be coded as a ‘forced’ answer. In the event that a participant expresses significant risk to themselves or others (e.g., suicidal ideation) in free-text comments, such as in the qualitative data, the lead investigator (EW), a registered clinical psychologist, will contact the participant to offer information on support services and referral options.

### Dissemination of Outcomes

Results will be disseminated in peer reviewed journals, *via* the media, online, and at academic conferences. A plain language summary of results from the study will be made available to participants upon request. Participants are advised of the process to request a plain language summary of the results in the Plain Language Statement.

### Future Research and Data Sharing

Participants are invited to provide optional consent to be contacted for future research, such as further follow-up beyond 6 months. This process would involve a new ethics application. Participants will also be invited to consent to their de-identified information being stored on public repositories for the purposes of data sharing. If consent is provided, participant data will be stored securely. All information about the study (including publication preprints, data access, and analytic code) will be available at https://osf.io/78g5t/.

#### Project Closure

At the conclusion of the study, recruitment materials, the project landing page, and online survey materials will be deactivated or removed. All data will remain securely stored on Deakin University servers. Information collected in this research project involves children who are under 18 years old, thus data will be kept until the youngest child turns 33 years of age.

### Recruitment Progress

The study was launched on the 8^th^ of April, 2020. As at the 26^th^ of April, 2,375 eligible participants had completed the baseline survey.

### Discussion

The COVID-19 pandemic presents significant risks to the mental health and wellbeing of Australian families. This project seeks to investigate the manifold impacts of the pandemic, including the impacts for families in regards to job loss, employment conditions, home-schooling, and unprecedented lifestyle changes associated with social distancing measures. Chronic stress and social isolation have potential risks for adult mental health, couple and family relationships, and children’s health and development (8–13). The novel contribution of the current study will be the repeated measures design, which will facilitate the tracking of changes in mental health over time in relation to the developing situation around the world.

This project is designed to provide timely information to government and communities on the mental health effects of the emerging COVID-19 crisis on Australian parents and children. This information can then be used to inform the development of assessment and screening tools to identify those parents, families, and children who may be most at risk. Furthermore, the findings of this research can guide health practitioners and policy makers regarding the factors that should be the focus of clinical and public health interventions to reduce risks of adult mental health, family breakdown, and child maladjustment when faced with such health crises in the future. Finally, the findings from this study can be used to develop practical information and advice for families in how to deal with such crises and create positive family environments to buffer against mental health problems, family dysfunction, and child maladjustment.

## Ethics Statement

The studies involving human participants were reviewed and approved by Deakin University Human Ethics Advisory Group (project number: HEAG-H 52_2020). The patients/participants provided their written informed consent to participate in this study.

## Author Contributions

All authors contributed to the conceptualization of the study, drafting of the study protocol, and selection of survey items/measures. EW drafted the manuscript, and together with TB, developed all study materials, including the online Qualtrics survey and the study adverts. EW, GK, JAM, LO, GY, CG, AM-W, ES, SE, MF-T, RC, DH, GM, JF, ST, AW, JT, TB, JL, PE, MS, and CO wrote or revised sections of the manuscript. All authors contributed to the article and approved the submitted version.

## Funding

The current study was funded through the Centre for Social and Early Emotional Development, a Strategic Research Centre at Deakin University.

## Conflict of Interest

The authors declare that the research was conducted in the absence of any commercial or financial relationships that could be construed as a potential conflict of interest.
